# Dapagliflozin Augmentation of Sacubitril/Valsartan Therapy in Patients With Heart Failure Following PCI‐Treated Acute Myocardial Infarction: A Meta‐Analysis of Clinical Efficacy and Safety

**DOI:** 10.1155/crp/1274068

**Published:** 2026-07-16

**Authors:** Tang Tang, Huimin Yu, Yun Zhang, Shuang Li, Mu Guo

**Affiliations:** ^1^ Department of Pharmacy, Shanghai Fourth People’s Hospital Affiliated Tongji University, Shanghai, China; ^2^ Department of Clinical Pharmacy, General Hospital of Eastern Theater Command, Nanjing, China; ^3^ Department of Cardiology, Shanghai Fourth People’s Hospital Affiliated Tongji University, Shanghai, China

**Keywords:** dapagliflozin, heart failure, meta-analysis, myocardial infarction, sacubitril/valsartan

## Abstract

**Purpose:**

To systematically review the efficacy and safety of dapagliflozin and sacubitril/valsartan (SV) in patients with heart failure (HF) following percutaneous coronary intervention (PCI)–treated acute myocardial infarction (AMI) and to provide evidence for clinical practice.

**Methods:**

Seven electronic databases were systematically searched from their inception to July 25, 2024. Following literature screening, data extraction, and risk of bias assessment, 14 randomized controlled trials involving 1310 patients were included. A meta‐analysis was subsequently conducted using RevMan 5.4 and Stata 18.

**Results:**

Meta‐analysis results showed that dapagliflozin combined with SV was superior to the control in improving left ventricular ejection fraction (LVEF) (WMD = 3.79, 95% CI: 2.70∼4.87; *p* < 0.001) and reducing N‐terminal pro‐brain natriuretic peptide (NT‐proBNP) levels (WMD = −83.49, 95% CI: −129.33∼−37.65; *p* = 0.0004). This combination also significantly reduced left ventricular end‐diastolic diameter (LVEDD) (WMD = −2.27, 95% CI: −3.64∼−0.90; *p* = 0.001), left ventricular end‐systolic diameter (LVESD) (WMD = −3.29, 95% CI: −3.98∼−2.59; *p* < 0.001), left ventricular remodeling index (LVRI) (WMD = −0.08, 95% CI: −0.10∼−0.06; *p* < 0.001), and left ventricular mass index (LVMI) (WMD = −8.84, 95% CI: −12.25∼−5.43; *p* < 0.001), while increasing the 6‐min walking distance (6MWD) (WMD = 51.74, 95% CI: 32.33∼71.14; *p* < 0.001). Compared with SV monotherapy, adding dapagliflozin reduced major adverse cardiovascular event (MACE) incidence (OR = 0.24, 95% CI: 0.15∼0.39; *p* < 0.001). However, no significant difference in adverse drug events (ADEs) was observed. Subgroup analyses revealed no significant differences in NT‐proBNP (WMD = −194.85, 95% CI: −396.53∼6.82; *p* = 0.06), LVEDD (WMD = −1.50, 95% CI: −3.90∼0.89; *p* = 0.22), or the incidence of ADE (OR = 1.26, 95% CI: 0.74∼2.14; *p* = 0.39) when the follow‐up duration was short (≤ 12 w). Additionally, significant heterogeneity was observed across studies for most outcomes. However, this heterogeneity was markedly reduced in the long‐term follow‐up subgroup (≥ 24 w), and the results were consistent with those of previous meta‐analyses.

**Conclusion:**

Current evidence suggests that early initiation of dapagliflozin combined with SV offers superior efficacy and cardiovascular benefits for patients with HF following PCI‐treated AMI. These benefits become increasingly evident with sustained treatment. Nevertheless, these conclusions warrant validation through more high‐quality evidence.

## 1. Introduction

Acute myocardial infarction (AMI) is a life‐threatening cardiovascular emergency with rapid onset, high mortality, and poor prognosis. Despite therapeutic advances, its mortality rate remains substantial [1]. Percutaneous coronary intervention (PCI) is a common treatment for AMI, which can recanalize occluded blood vessels and restore myocardial perfusion, thus markedly improving patient survival [2]. However, approximately one‐quarter of patients still develop acute or chronic heart failure (HF) postprocedure [3, 4], which may be related to imbalance of inflammatory factors, cardiomyocyte apoptosis or necrosis, and ventricular remodeling [5].

Sacubitril/valsartan (SV) is an angiotensin receptor neprilysin inhibitor (ARNI). It combines an angiotensin II receptor blocker and a neprilysin inhibitor, serving as a first‐line drug for HF [6]. Research has demonstrated that SV can significantly suppress postinfarction cardiomyocyte hypertrophy and fibrosis, increase capillary density, and thereby improve left ventricular ejection fraction (LVEF) [7, 8]. For post‐AMI patients, initiating SV early provides greater benefit than conventional angiotensin‐converting enzyme inhibitors (ACEIs)/angiotensin receptor blocker (ARB) therapy by lowering the risk of HF hospitalization, enhancing cardiac function, and promoting reverse left ventricular remodeling [9]. Dapagliflozin is a sodium‐glucose cotransporter 2 inhibitor (SGLT‐2i) that was originally developed as a hypoglycemic agent. It exerts cardioprotective, antioxidant, and anti‐inflammatory effects by downregulating myocardial NF‐κB expression, inhibiting systemic proinflammatory biomarkers, and reducing lipid peroxidation [10]. Several guidelines have indicated that dapagliflozin can reduce all‐cause and cardiovascular mortality in patients with HF with reduced ejection fraction [6, 11]. Additionally, it can relieve HF symptoms, improve physical function, and enhance quality of life. Recently, many researchers have focused on the potential benefits of dapagliflozin in patients with AMI [12, 13]. However, robust clinical evidence regarding the efficacy and safety of combining dapagliflozin and SV in patients with HF following PCI‐treated AMI remains lacking. Therefore, we conducted a systematic review and meta‐analysis to compare the clinical outcomes associated with dapagliflozin and SV in these patients.

## 2. Methods

The study protocol was registered with PROSPERO (CRD42024569210). This meta‐analysis was performed in compliance with the Preferred Reporting Items for Systematic Reviews and Meta‐Analyses (PRISMA) guidelines.

### 2.1. Search Strategy

Databases including PubMed, CBM, Cochrane Library, OVID Medline, CNKI, Wan Fang Data, and VIP were searched for relevant studies from inception to July 25, 2024. Relevant literature, such as reports and conference articles, was also searched. The search terms were as follows: “acute myocardial infarction,” “AMI,” “heart failure,” “dapagliflozin,” “SGLT‐2i,” “sodium‐glucose transporter 2 inhibitors”; “sacubitril valsartan,” “LCZ696,” “ARNI,” “Entresto,” and “angiotensin receptor neprilysin inhibitor.”

### 2.2. Inclusion and Exclusion Criteria

The inclusion criteria were as follows: (I) studies involving patients with HF following PCI‐treated AMI, (II) studies in which the experimental group received SV plus dapagliflozin and the control group received SV alone; and (III) RCTs. The exclusion criteria were as follows: (I) studies lacking an appropriate control group; (II) animal studies; (III) studies focusing on HF not secondary to PCI‐treated AMI; (IV) non‐RCTs; and (V) studies that did not report efficacy and safety‐related data. All literature searches were conducted independently by two reviewers, with discrepancies resolved through discussion or by consultation with a third reviewer.

### 2.3. Data Extraction and Outcomes

Two reviewers independently extracted the following data from each of the included studies: first author, publication year of study, country, characteristics of patient (age and reperfusion time since AMI), and intervention details (grouping, sample size, drugs, and intervention duration). The primary outcomes of this study included left ventricular remodeling parameters, namely LVEF, left ventricular end‐diastolic diameter (LVEDD), left ventricular end‐systolic diameter (LVESD), left ventricular remodeling index (LVRI), and left ventricular mass index (LVMI), as well as major adverse cardiovascular event (MACE). The secondary outcomes included N‐terminal pro‐brain natriuretic peptide (NT‐proBNP) and 6‐min walking distance (6MWD). The safety outcome was the incidence of adverse drug events (ADEs).

### 2.4. Quality Assessment

Two independent reviewers assessed the risk of bias of all included studies using the RCT risk‐of‐bias assessment tool recommended in the Cochrane Handbook, and the results were cross‐checked [14].

### 2.5. Statistical Analysis

Statistical analyses were performed using RevMan 5.4 and Stata 18.0 software. Continuous variables were presented as mean ± SD. Treatment effects for continuous outcomes were estimated using the weighted mean differences (WMDs) with 95% confidence intervals (CIs), while dichotomous variables were expressed as odds ratios (ORs) with 95% CIs. Heterogeneity between studies was assessed by the *χ* ^2^ test, and its magnitude was quantified using the *I* ^2^ statistic. When *p* ≥ 0.1and *I* ^2^ ≤ 50%, heterogeneity among studies was considered low, and a fixed‐effect model was used for meta‐analysis. Otherwise, a random‐effects model was used. Then, subgroup analysis and sensitivity analysis were conducted if significant heterogeneity was present. Publication bias was assessed using funnel plots when ten or more studies were included in the meta‐analysis.

## 3. Results

### 3.1. Search Results and Study Characteristics

**FIGURE 1:**
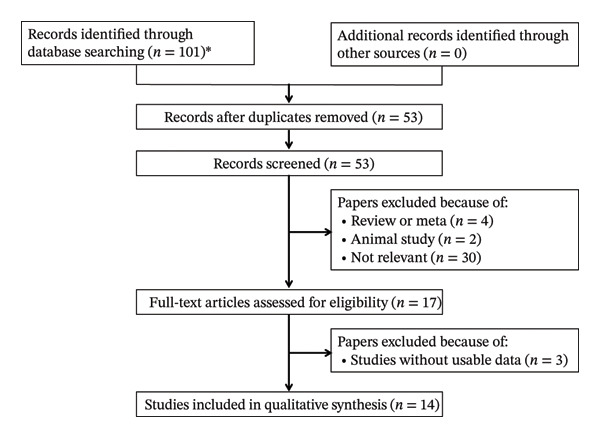
Flowchart for literature screening (PRISMA flow diagram). ^∗^PubMed (*n* = 7); CBM (*n* = 9); the Cochrane Library (*n* = 10); OVID Medline (*n* = 3); CNKI (*n* = 23); Wan Fang data (*n* = 21); VIP (*n* = 28).

The study selection process is shown in Figure 1. Initially, 101 relevant studies were identified. After removing duplicates and applying screening criteria, 14 RCTs [15–28] were ultimately eligible for our study. Table 1 provides an overview of the characteristics of these RCTs, all of which were conducted in China and included a total of 1310 participants. Among the included studies, 655 participants were assigned to the experimental group and 655 to the control group. Lv’s study [24] had three therapy groups, two of which were included according to the inclusion criteria. In terms of intervention duration, the shortest was 4 weeks, and the longest was 36 weeks.

**TABLE 1:** The characteristics of the included studies.

Study	Country	*n* (T/C)	Age (T/C, years old)	Reperfusion time (h)	Intervention and control	Intervention duration (weeks)	Outcomes
Wang 2022 [15]	China	36/36	63.28 ± 6.50/63.15 ± 6.74	NR	Dapagliflozin and SV/SV	12	1, 2, 3, 6, 7, 8, 9
Sheng 2022 [16]	China	60/60	64.59 ± 9.10/62.78 ± 6.53	6.47 ± 2.28/6.22 ± 2.14	Dapagliflozin and SV/SV	24	1, 2, 3, 4, 5, 6, 8, 9
Pan 2022 [17]	China	110/103	67.75 ± 9.14/66.80 ± 10.05	6.49 ± 2.68/6.78 ± 2.35	Dapagliflozin and SV/SV	36	1, 2, 3, 4, 7, 8, 9
Su 2023 [18]	China	50/50	58.90 ± 9.39/58.22 ± 8.10	NR	Dapagliflozin and SV/SV	12	1, 2, 3, 6, 7, 8, 9
Liu 2023 [19]	China	30/30	61.8 ± 12.3/61.2 ± 11.6	7.1 ± 3.6/6.4 ± 3.2	Dapagliflozin and SV/SV	12	2, 3, 4, 5, 9
Wu 2024 [20]	China	53/54	64.13 ± 8.62/64.95 ± 8.14	NR	Dapagliflozin and SV/SV	12	1, 2, 3, 6, 7, 8, 9
Li 2024 [21]	China	46/46	63.24 ± 8.37/61.37 ± 7.25	4.11 ± 0.63/4.27 ± 0.61	Dapagliflozin and SV/SV	24	1, 2, 4, 6, 7, 8, 9
Gao 2024 [22]	China	30/33	74.77 ± 5.01/75.14 ± 4.63	NR	Dapagliflozin and SV/SV	12	1, 2, 3, 6, 7, 8, 9
Zheng2024 [23]	China	36/36	62.42 ± 4.31/62.35 ± 4.28	6.47 ± 2.05/6.51 ± 2.12	Dapagliflozin and SV/SV	12	1, 2, 3, 7, 9
Lv 2024 [24]	China	45/48	67.11 ± 5.35/68.23 ± 7.42	NR	Dapagliflozin and SV/SV	24	1, 2, 3, 6, 7
Duan 2024 [25]	China	30/30	36.58 ± 3.17/37.62 ± 3.51	NR	Dapagliflozin and SV/SV	24	1, 2, 3, 4, 5, 6
Liu 2024 [26]	China	45/45	63.67 ± 6.28/63.54 ± 6.05	NR	Dapagliflozin and SV/SV	4	1, 2, 7
Tang 2024 [27]	China	40/40	64.18 ± 5.20/64.58 ± 5.17	4.05 ± 0.58/4.02 ± 0.55	Dapagliflozin and SV/SV	12	1, 3, 4, 9
Liang 2024 [28]	China	30/30	63 ± 12.2/64 ± 12.4	5.3 ± 1.4/5.3 ± 1.4	Dapagliflozin and SV/SV	24	1, 2, 3, 6

*Note:* Outcomes: 1, LVEF; 2, LVEDD; 3, LVESD; 4, LVRI; 5, LVMI; 6, MACE; 7, NT‐proBNP; 8, 6MWD; 9, ADE.

Abbreviation: NR, not reported.

### 3.2. Risk of Bias in the Included Studies

**FIGURE 2:**
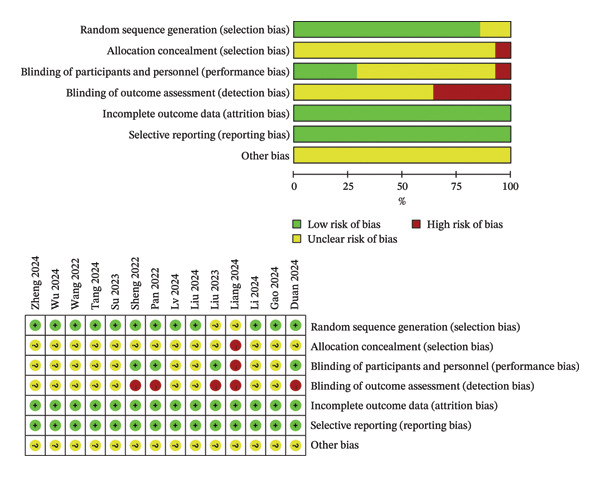
Summary of the quality assessment.

The methodological quality of all included trials was assessed using the Cochrane Collaboration risk of bias tool Figure 2. The randomization method was not clearly described in the studies by Liu [19] and Liang [28]. Pan [17] reported generating random numbers by coin tossing, while Sheng [16] mentioned randomization but did not specify the method. The remaining studies [15, 18, 20–27] utilized random number table for randomization. Regarding allocation concealment, the study of Liang [28] was open‐label, whereas the other studies did not report this information. In terms of blinding, three studies [16, 17, 25] were single‐blind, and one study [19] was double‐blind.

### 3.3. Primary Outcomes

#### 3.3.1. Left Ventricular Remodeling Parameters

The meta‐analysis results of left myocardial remodeling parameters are shown in Figure 3.

FIGURE 3Forest plots of left ventricular remodeling parameters. ((A): LVEF; (B): LVEDD; (C): LVESD; (D): LVRI; (E): LVMI.).

**Figure:**
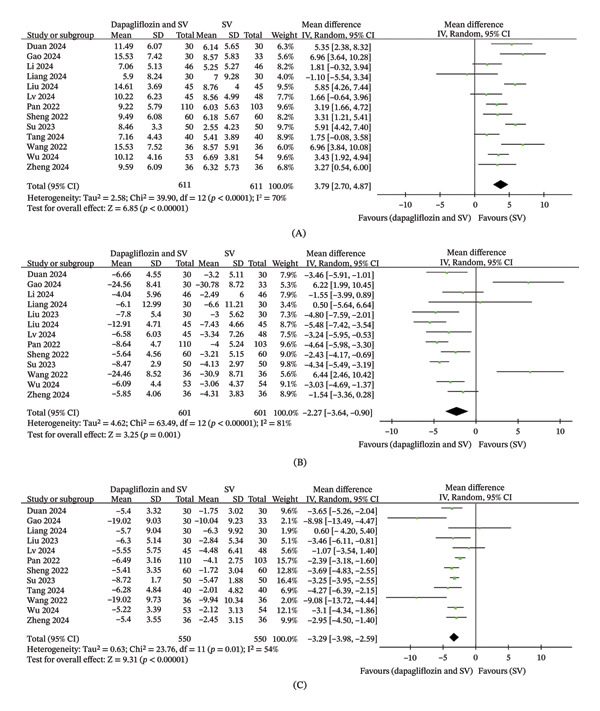

**Figure:**
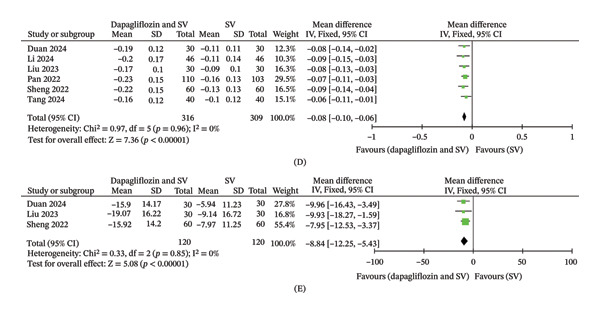

A total of 13 studies [15–18, 20–28] compared the change in LVEF after treatment between the two groups. There was significant heterogeneity across the studies (*I* ^2^ = 70%, *p* < 0.001). Therefore, a random‐effects model was used. Meta‐analysis revealed that the improvement in LVEF was significantly greater in the combination treatment group (WMD = 3.79, 95% CI: 2.70∼4.87; *p* < 0.001).

A total of 13 trials [15–26, 28] reported the outcome of LVEDD. Significant heterogeneity was noted among the included studies (*I* ^2^ = 81%, *p* < 0.001), and a random‐effects model was used. The results indicated that combination treatment was superior in reducing LVEDD (WMD = −2.27, 95% CI: −3.64∼−0.90; *p* = 0.001).

A total of 12 trials [15–20, 22–25, 27, 28] reported the outcome of LVESD. Meta‐analysis results using a random‐effects model (*I* ^2^ = 54%, *p* = 0.01) showed that combination treatment reduced LVESD significantly (*WMD* = −3.29, 95% CI: −3.98∼−2.59; *p* < 0.001).

Six studies [16, 17, 19, 21, 25, 27] reported the outcome of LVRI. A fixed‐effect model was used (*I* ^2^ = 0%, *p* = 0.96). Meta‐analysis results showed that combination treatment reduced LVRI significantly (*WMD* = −0.08, 95% CI: −0.10∼−0.06; *p* < 0.001).

Three studies [16, 19, 25] reported the outcome of LVMI. A fixed‐effect model was used (*I* ^2^ = 0%, *p* = 0.85). Meta‐analysis results showed that combination treatment reduced LVMI significantly (WMD = −8.84, 95% CI: −12.25∼−5.43; *p* < 0.001).

#### 3.3.2. MACE Outcome

**FIGURE 4:**
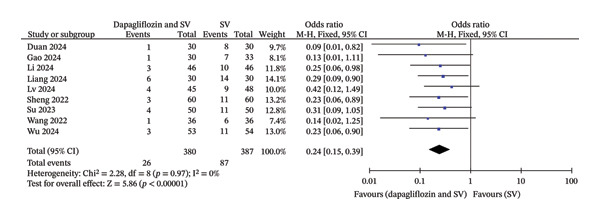
Forest plot of MACE outcomes.

MACE was defined as recurrent angina, nonfatal myocardial infarction (MI), HF rehospitalization, or cardiac death. As shown in Figure 4, studies [15, 16, 18, 20–22, 24, 25, 28] compared the incidence of MACE after therapy. With no significant heterogeneity observed (*I* ^2^ = 0%, *p* = 0.97), a fixed‐effect model was used. The meta‐analysis showed that the incidence of MACE in the experimental group was significantly lower than that in the control group (OR = 0.24, 95% CI: 0.15∼0.39; *p* < 0.001).

### 3.4. Secondary Outcomes

**FIGURE 5:**
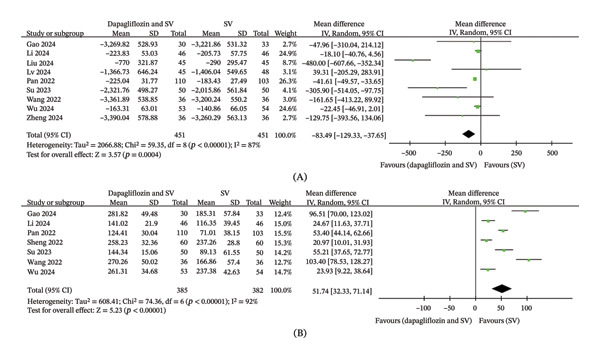
Forest plots of NT‐proBNP levels and 6MWD. ((A): NT‐proBNP; (B): 6MWD).

The meta‐analysis results of NT‐proBNP level and 6MWD are shown in Figure 5.

A total of 9 studies [15, 17, 18, 20–24, 26] compared the change of NT‐proBNP level between the two groups after treatment. There was significant heterogeneity across the studies (*I* ^2^ = 87%, *p* < 0.001), and a random‐effects model was used. Meta‐analysis showed that the NT‐proBNP level decreased significantly in the combination treatment group (WMD = −83.49, 95% CI: −129.33∼−37.65; *p* = 0.0004).

Seven studies [15–18, 20–22] reported the outcome of 6MWD. A random‐effects model was used (*I* ^2^ = 92%, *p* < 0.001). Meta‐analysis results showed that combination treatment significantly increased 6MWD compared with control therapy (WMD = 51.74, 95% CI: 32.33∼71.14; *p* < 0.001).

### 3.5. Safety Outcome

**FIGURE 6:**
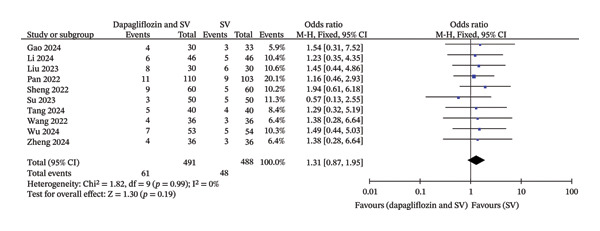
Forest plot of the ADE outcome.

The incidence of ADE was compared between two groups based on 10 studies to assess the safety of dapagliflozin and SV [15–23, 27] (Figure 6). No significant heterogeneity was observed among the included studies (*I* ^2^ = 0%, *p* = 0.99), so a fixed‐effect model was adopted. The results showed no statistically significant difference in the ADE incidence rate between the two groups (OR = 1.31, 95% CI: 0.87∼1.95; *p* = 0.19). The reported ADEs comprised kidney injury, hypotension, hypoglycemia, urinary tract infection, angioneurotic edema, gastrointestinal reactions, hyperkalemia, and ketoacidosis.

### 3.6. Subgroup Analyses

Subgroup analyses were performed based on different follow‐up times (Table 2), which were categorized into a short‐term group (≤ n12 weeks) and a long‐term group (≥ 24 weeks).

**TABLE 2:** Subgroup analyses of outcomes at different follow‐time.

Follow‐time	Number of studies	Heterogeneity		Analysis model	WMD/OR (95% CI)^∗^	*p*
		*I* ^2^ (%)	*p*			
Short‐term group LVEF	7	73	0.001	Random‐effects	4.72 (3.25, 6.18)	< 0.001
LVEDD	7	89	< 0.001	Random‐effects	−1.50 (−3.90, 0.89)	0.22
LVESD	7	54	0.04	Random‐effects	−3.81 (−4.82, −2.80)	< 0.001
LVRI	2	0	0.59	Fixed‐effect	−0.07 (−0.11, −0.03)	0.0002
LVMI	1	—	—	Random‐effects	−9.93 (−18.27, −1.59)	0.02
MACE	4	0	0.87	Fixed‐effect	0.22 (0.10, 0.48)	0.0001
NT‐proBNP	6	91	< 0.001	Random‐effects	−194.85 (−396.53, 6.82)	0.06
6MWD	4	93	< 0.001	Random‐effects	68.61 (31.54, 105.68)	0.0003
ADE	7	0	0.97	Fixed‐effect	1.26 (0.74, 2.14)	0.39
Long‐term group LVEF	6	38	0.15	Fixed‐effect	2.75 (1.85, 3.65)	< 0.001
LVEDD	6	39	0.14	Fixed‐effect	−3.36 (−4.21, −2.51)	< 0.001
LVESD	5	51	0.08	Random‐effects	−2.74 (−3.75, −1.73)	< 0.001
LVRI	4	0	0.92	Fixed‐effect	−0.08 −0.10, −0.05)	< 0.001
LVMI	2	0	0.62	Fixed‐effect	−8.62 (−12.36, −4.88)	< 0.001
MACE	5	0	0.83	Fixed‐effect	0.26 (0.14, 0.47)	< 0.001
NT‐proBNP	3	51	0.13	Random‐effects	−32.37 (−52.60, −12.13)	0.002
6MWD	3	9	< 0.001	Random‐effects	33.28 (11.44, 55.11)	0.003
ADE	3	0	0.78	Fixed‐effect	1.37 (0.74, 2.57)	0.32

*Note:* MACE and ADE were expressed as ORs with 95% CIs.

^∗^LVEF, LVEDD, LVESD, LVRI, LVMI, NT‐proBNP, and 6MWD were estimated using WMD with 95% CIs.

In the short‐term group, significant heterogeneity across studies was observed for the outcomes of LVEF, LVEDD, LVESD, NT‐proBNP, and 6MWD. The LVMI outcome was reported in only one trial. Accordingly, a random‐effects model was used for these outcomes, and a fixed‐effect model was chosen for the outcomes of LVRI, MACE, and ADE. The experimental group showed better performance than the control group for most of these outcomes. However, no significant differences were observed between the two groups for NT‐proBNP (WMD = −194.85, 95% CI: −396.53∼6.82; *p* = 0.06) and LVEDD (WMD = −1.50, 95% CI: −3.90∼0.89; *p* = 0.22). Regarding safety, there was no significant difference in the incidence of ADE between the two groups (OR = 1.26, 95% CI: 0.74∼2.14; *p* = 0.39).

In the long‐term group, heterogeneity was significantly reduced for most outcomes. The substantial heterogeneity observed in the 6MWD across studies may have stemmed from differences in protocol implementation among research centers [29]. A random‐effects model was applied to the outcomes of NT‐proBNP, LVESD, and 6MWD, while a fixed‐effect model was chosen for the other outcomes. The meta‐analysis indicated that the experimental group yielded superior efficacy without a significant increase in safety risks relative to the control group, aligning with the earlier findings.

### 3.7. Sensitivity Analysis

Sensitivity analyses were conducted by sequentially eliminating one study at a time and repeating meta‐analysis on the remaining studies. After the exclusion of Liu’s study [26], there was a substantial reduction in heterogeneity for the NT‐proBNP outcome (*I* ^2^ = 48%, *p* = 0.06). However, this did not alter the overall conclusion of the meta‐analysis (WMD = −38.02, 95% CI: −45.19∼−30.86; *p* < 0.001), indicating that the results were stable.

### 3.8. Publication Bias

**FIGURE 7:**
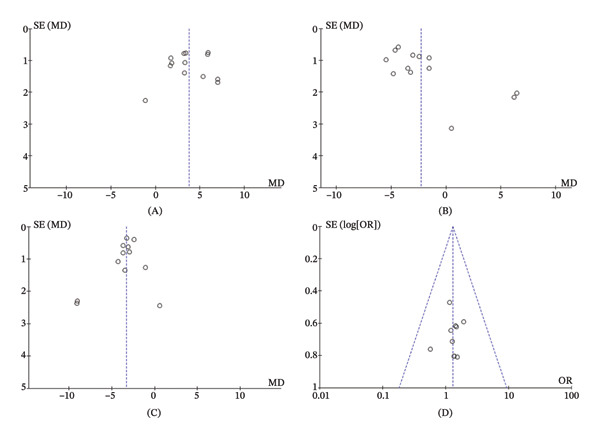
Funnel plots of LVEF, LVEDD, LVESD, and ADE. ((A): LVEF; (B): LVEDD; (C): LVESD; (D): ADE).

Funnel plot and Egger’s test were performed to evaluate publication bias when the number of included studies was ≥ 10. As shown in Figure 7, the symmetric funnel plot indicated no significant publication bias for the occurrence of ADE. In contrast, the funnel plots for LVEF, LVEDD, and LVESD showed some asymmetry, raising the possibility of publication bias. However, Egger’s regression test yielded nonsignificant results for LVEF (*p* = 0.5022), LVEDD (*p* = 0.2998), and LVESD (*p* = 1.0000), indicating no statistically significant publication bias for these outcomes.

## 4. Discussion

The persistence or exacerbation of the inflammatory response following AMI significantly contributes to left ventricular remodeling and the development of HF [30]. Intramyocardial hemorrhage following MI leads to iron deposition in cardiomyocytes, which triggers a proinflammatory response and ultimately contributes to adverse left ventricular remodeling [31]. The BRIGHT study reported a 14.3% incidence of HF among patients with PCI‐treated AMI at admission [32]. The occurrence of HF after AMI is associated with increased short‐term and long‐term mortality. Notably, AMI patients with HF exhibited a significantly higher risk of 1‐year postdischarge mortality compared to those without HF [33, 34]. SV inhibits neprilysin and increases the levels of cardiovascular protective factors, and its therapeutic efficacy in patients with HF following PCI‐treated AMI has been well established [7, 35]. Furthermore, dapagliflozin has been shown to reduce the incidence of MACE and the risk of all‐cause and cardiovascular mortality in patients with HF with reduced ejection fraction [36, 37]. Therefore, this study aims to investigate the efficacy and safety of early initiation and sustained use of dapagliflozin added to SV therapy in patients with HF following PCI‐treated AMI.

These meta‐analysis results showed that during the early treatment stage, dapagliflozin combined with SV demonstrated significant advantages in outcomes such as LVEF, LVESD, LVRI, LVMI, and 6MWD, while also reducing the incidence of MACE. However, no significant differences were observed in NT‐proBNP and LVEDD levels between the two groups. Considerable heterogeneity was observed among the included studies for most outcomes. However, as the duration of follow‐up increased, the treatment effects across trials became more consistent. The combination therapy of dapagliflozin and SV demonstrated superior efficacy compared to SV monotherapy. For safety outcome, there was no significant difference compared with SV.

SGLT‐2i has been proven to provide consistent benefits across the full spectrum of ejection fraction in HF [38]. Consequently, they are now widely used and, together with ACEI (or ARB or ARNI), beta‐blockers, and mineralocorticoid receptor antagonists, constitute the cornerstone of HF therapy [39, 40]. Current therapeutic guidelines highlight that a primary goal of HF management is to improve postdischarge clinical outcomes, specifically by reducing readmissions and mortality [11, 39, 40]. Recently, the application of SGLT‐2i for HF has been progressively extended to the post‐MI population. The EMPACT‐MI trial assessed empagliflozin in patients with AMI presenting with either a newly reduced LVEF < 45% or clinical signs of congestion. While empagliflozin significantly lowered the risks of first and total HF hospitalizations, it failed to achieve a significant reduction in the primary composite endpoint of time to first HF hospitalization or all‐cause death [41]. Additionally, the efficacy of dapagliflozin in reducing the primary composite endpoint of cardiovascular death or HF hospitalization among patients following AMI is not yet fully established [41, 42]. However, a meta‐analysis indicated that SGLT‐2i (empagliflozin and dapagliflozin) reduces the relative risk of sudden cardiac death in patients with Type 2 diabetes, HF, or chronic kidney disease, thereby expanding the spectrum of cardiovascular benefits associated with this drug class. This protective effect may be attributed to the ability of SGLT‐2i′s to improve myocardial ionic homeostasis, modulate autonomic balance, and reduce myocardial fibrosis and oxygen consumption [43]. The findings of this study demonstrate that the adjunctive use of dapagliflozin reduces the incidence of MACE in patients with HF following PCI‐treated AMI, providing additional support for the cardiovascular benefits of dapagliflozin in the post‐MI population. Furthermore, the sequence of medication initiation in guideline‐directed medical therapy for HF may influence clinical outcomes. The early introduction of dapagliflozin, in particular, has the potential to reduce the incidence of the composite endpoint of HF hospitalization or cardiovascular death [44]. Our study demonstrates that in patients with HF following PCI‐treated AMI, initiating dapagliflozin early yields greater clinical benefits compared to SV alone. Although this benefit may vary among patients during the early treatment phase, it becomes increasingly evident as the duration of therapy extends.

Dapagliflozin induces a state of negative energy balance by promoting urinary glucose excretion. This fasting‐like metabolic state promotes the synthesis of ketone bodies, which possess inherent antioxidant and anti‐inflammatory properties [45]. These anti‐inflammatory and antioxidant effects are mediated by multiple mechanisms. In addition to the previously mentioned NF‐κB pathway [10], dapagliflozin also attenuates tissue inflammation and exerts organ‐protective effects by inhibiting key inflammatory signaling pathways, including the STING pathway and the NLRP3 inflammasome [46, 47]. During ischemia/reperfusion, dapagliflozin significantly mitigated lipid peroxidation, oxidative stress, iron overload, and ferroptosis, thereby markedly ameliorating myocardial injury and cardiac function [48]. However, the benefits of combining dapagliflozin with SV in early treatment may vary among patients. S Cortellino’s research has found that a fasting‐mimicking diet has to be administered over a sufficient dietary cycle to observe its effects on tumor growth and immune cells [49]. Similarly, the cardiovascular benefits conferred by dapagliflozin through a fasting‐like metabolic state may also require a certain duration of treatment to become evident. With increasing of follow‐up time, the benefits of dapagliflozin become more significant for patients. The clinical indicators, including left myocardial remodeling parameters, NT‐proBNP, 6MWD, and MACE rate, were significantly improved with the combination of dapagliflozin and SV compared with SV alone.

Given the relatively small sample size of this analysis, further research is needed to validate the cardiovascular benefits of dapagliflozin in HF patients following PCI‐treated AMI and to explore both the underlying mechanisms and the impact of treatment duration on its therapeutic efficacy.

## 5. Conclusion

Current evidence indicates that in patients with HF following PCI‐treated AMI, early addition of dapagliflozin to the treatment regimen can reduce HF‐related biomarker levels, reverse and delay ventricular remodeling, improve quality of life, and provide cardiovascular benefits. Furthermore, the cardiovascular benefits of dapagliflozin become more consistent with longer treatment duration, while demonstrating a favorable safety profile. Therefore, the early initiation and long‐term use of dapagliflozin in patients with HF following PCI‐treated AMI show significant clinical promise. However, as the included studies were all conducted in Chinese populations, further clinical trials are needed to validate these findings.

## Author Contributions

Mu Guo and Shuang Li: conception and design. Yun Zhang: provision of study materials. Tang Tang and Huimin Yu: data curation and formal analysis. Tang Tang: original manuscript.

## Funding

This study was funded by the Scientific Research Foundations of Shanghai Fourth People’s Hospital affiliated Tongji University (Nos. Sykyqd06001, sykygd13101, and SY‐XKZT‐2025‐1019).

## Disclosure

All authors reviewed and gave final approval of the manuscript.

## Conflicts of Interest

The authors declare no conflicts of interest.

## Data Availability

The data supporting the meta‐analysis are from previously reported studies and datasets, which have been cited.
